# Alcohol Consumption and Risk of Liver Fibrosis in People Living With HIV: A Systematic Review and Meta-Analysis

**DOI:** 10.3389/fimmu.2022.841314

**Published:** 2022-03-18

**Authors:** Hang Lyu, Haotong Tang, Yizhi Liang, Shaoli Huang, Yuyu Wang, Wenyan Huang, Yi Zhou

**Affiliations:** ^1^ Department of HIV Prevention, Zhuhai Center for Disease Control and Prevention, Zhuhai, China; ^2^ Faculty of Medicine, Macau University of Science and Technology, Macao, Macao SAR, China; ^3^ West China School of Public Health, Sichuan University, Chengdu, China; ^4^ Zhejiang University-University of Edinburgh Institute, Zhejiang University, Jiaxing, China; ^5^ School of Medicine, Jinan University, Guangzhou, China

**Keywords:** HIV, alcohol, liver fibrosis, cross-sectional study, HIV/HCV co-infection

## Abstract

**Objectives:**

It is unclear if a high level of alcohol consumption is a risk factor for liver fibrosis for people living with HIV (PLWH). This study systematically summarizes the risk relationship between different alcohol consumption and the incidence of liver fibrosis among PLWH.

**Methods:**

We identified potential studies by searching the PubMed, Embase, Web of Science Library, and CNKI databases up to September 26th, 2021. Observation studies in PLWH that evaluated the relationship between alcohol consumption and the risk of liver fibrosis and estimated the effect of alcohol with pooled odds ratios (pooled ORs) and 95% confidence intervals (CIs) were included.

**Results:**

There were total 15 studies included in data analysis. Three studies were set up as cohort studies and the other twelve were cross-sectional studies. Our study was based on 22,676 individuals and 2,729 liver fibrosis cases from 15 studies. Alcohol abuse is a significant risk factor of liver fibrosis (pooled OR = 2.25, 95% CI: 1.59-3.17, p < 0.05) among PLWH. Daily alcohol consumption > 50 g can elevate the risk of liver fibrosis (pooled OR = 3.10, 95% CI: 2.02-4.73, p < 0.05) among PLWH. However, high-risk alcohol consumption determined by AUDIT-C (AUDIT-C ≥ 4) had little or no effect on subsequent liver fibrosis risk. Further, alcohol consumption > 50 g is also a risk factor to liver fibrosis in PLWH co-infected with HCV (pooled OR = 2.48, 95% CI: 1.62-3.80, p < 0.05) and in HIV mono-infected (pooled OR = 1.85, 95% CI: 1.00-3.43, p < 0.05).

**Conclusion:**

Alcohol consumption is associated with an increased risk of liver fibrosis in PLWH. HCV co-infection with alcohol abuse could possibly induce a higher risk of liver fibrosis than HIV mono-infected patients.

**Systematic Review Registration:**

PROSPERO, identifier (CRD42021272604).

## Highlights

**What Is the Current Knowledge?**

Alcohol is a driving force of liver fibrosis and cirrhosis in general people.

Due to the continuous destruction of the immune system, PLWH are more prone to develop liver fibrosis and other liver diseases.

**What Is New Here?**

High alcohol consumption is associated with increased liver fibrosis risk in PLWH, especially HIV/HCV co-infected patients.

Inconsistent definitions of alcohol exposure can lead to different conclusions.

## Introduction

Liver fibrosis is one of the most common liver diseases (1). Liver fibrosis (including cirrhosis) is a leading cause of death, affecting more than 600,000 people each year (2). According to 2015 data, the prevalence of liver fibrosis in America is about 0.27%, which is equivalent to 633,323 adults (3). In China, it is estimated that more than one-fifth of the population is affected by some form of liver disease (4), and more than 7 million (0.5% of the total Chinese population) live with liver cirrhosis, from which stem 460,000 new cases of liver cancer per year in China (5). In chronic liver disease, fibrosis develops as the result of an imbalance between the new deposition of extracellular matrix (ECM) and its resorption, representing the wound healing response of the liver to repeated injury (6). If liver fibrosis cannot be treated and intervened in time, it will progress to liver cirrhosis or even liver cancer, which is a heavy disease burden also in Africa (7). Among people living with HIV (PLWH), the incidence of hepatic fibrosis is increasing (8). The reasons include the use of antiretroviral drugs (9), simultaneous infection with HBV or HCV (10), decreased immune function (11), and drug abuse (12), especially alcohol.

Alcohol is a major risk factor for liver fibrosis in general, and for PLWH in particular (13). Alcohol consumption can complicate HIV infection and lead to comorbidities (14). Alcohol use negatively affects HIV disease progression through multiple mechanisms, including transmission, viral replication (15), immunity system (16), and therapeutic efficacy (17). Among those using PI-based regimens, hazardous alcohol drinkers had significantly higher liver fibrosis (18). HIV can affect the immune system, thereby affecting the function of vital organs including the liver, and then induce fibrosis generation (19). As liver fibrosis is a liver wound healing reaction, which often occurs in alcoholic hepatitis, and is characterized by excessive production and accumulation of fibrotic matrix, mainly type I collagen (20). Previous study (21) reported that whether under acute or chronic alcohol stimulation, the metabolic process of ethanol produces a large amount of reactive oxygen species (ROS), causing mitochondrial damage and fat oxidative degeneration, which is important pathogenesis of the alcoholic liver disease. At the same time, ROS can also inhibit the differentiation ability of cells and the repairability of damaged tissues, increase the synthesis of collagen fibers, and accelerate liver fibrosis formation, leading to liver fibrosis’s progress (22, 23). Researchers believe that cytokines mediate alcohol-induced liver fibrosis, the most important being the pro-fibrotic factor *TGF-β (* 24). Also, others verified (25) that drinking leads to intestinal microecological disorders, and the intestinal epithelium’s integrity is destroyed, leading to the influx of immune cells into the liver, forming intestinal endotoxemia and ultimately liver fibrosis. However, there is also literature that indicates alcohol intake does not affect liver disease or liver fibrosis (26, 27).

In the present study, we defined specific liver stiffness cutoffs for alcohol-induced liver fibrosis in PLWH, with particular consideration given to the effects of study design, alcohol determination, and HCV co-infection, and other subgroups where data were available.

## Methods

### Search Strategy and Selection Criteria

Eligible records were systematically retrieved in four authoritative databases, including PubMed, Web of Science, Embase and CNKI databases, until September 26th, 2021. We obtained relevant articles regarding alcohol-related liver fibrosis in PLWH, with the following keywords including “Alcohol (or Alcohol drinking or binge drink or ethanol) and HIV (or human immunodeficiency virus) and fibrosis (or cirrhosis)”. Inclusion criteria were as follows:1) Cohort, case-control and cross-sectional studies examining the association between average alcohol consumption and liver fibrosis in PLWH. 2) Data included OR RR for alcohol and liver fibrosis. 3) Data for liver fibrosis was adjusted for liver biopsy or transient elastography or APRI, FIB-4 index, and can be transient to fibrosis stage. 4) Alcohol consumption was quantitative by average alcohol consumption per day or AUDIT-C categories or others. 5) More than 40 cases of liver fibrosis occurred. Studies published using data from the same population, studies with incomplete data or unclear outcomes, and the type of literature is review, meta-analysis, or conference abstract, etc. on were excluded.

### Data Extraction

Two investigators (H.L. and Y.L.) independently gathered the information from each qualified publication. Data extraction of each qualified article was as follows: first author and publication year, country, number of total participants, number of observed liver fibrosis cases among participants, sex, age (range, mean or median), whether co-infected with HCV, alcohol exposure assignment, liver fibrosis outcome assignment and adjustments. Two investigators reached a consensus on the basis of discussion when they had different opinions.

### Exposure and Outcome Assessment

The Alcohol Use Disorders Identification Test–Consumption questionnaire (AUDIT-C), which was firstly published in 1989 by WHO (28), was developed for use in a primary care setting and is now used as a screening toll in many clinical and research settings. The daily alcohol consumption was determined by asking participants to retrospectively recount the number of drinks consumed on each day (29). Both daily alcohol consumption and AUDIT-C are validated and widely used screening tools for alcohol use and abuse and are subject to both recall and stigma biases (30, 31). For alcohol consumption, we converted reported alcohol intake categories in primary studies into an average of pure alcohol in grams per day (g/day) using the midpoints (mean or median) of reported drinking group categories. The majority of studies defined “high-risk alcohol consumption” as alcohol intake > 50 g/day for more than 30 days. The “low-risk alcohol consumption” was defined as alcohol intake < 50 g/day. Some studies used the AUDIT-C score to assess alcohol use. The AUDIT-C is collected as part of self-administered standardized clinical assessments which were introduced into routine care at CNICS sites. But the cut-off of different studies was different according to AUDIT-C score. We combined the results of a large number of literature and found “current hazardous drinking” or “high-risk alcohol consumption” can be defined as AUDIT-C ≥ 4. Nonhazardous drinking also can be known as “low risk alcohol consumption” can be defined as AUDIT-C score < 4.

There are three methods to assess in clinical study for liver fibrosis: liver biopsy, transient elastography (TE), and fibrosis score. Although liver biopsy is the “gold standard” for liver fibrosis assessment, it is not often used in research because of traumatic. In the study assess liver fibrosis by biopsy, liver fibrosis can be stratified into three categories: absent or mild (Metavir stage F0 or F1), moderate (F2), and severe (F3 or F4) (32). Transient elastography is another method to quantify the liver fibrosis stage, this method is simple and feasible, but the doctor’s technical requirements are very high (33). According to the articles, patients were classified into four fibrosis groups depending on the TE values obtained: mild or no fibrosis (TE < 7.2 kPa, F0 or F1), significant fibrosis (TE 7.2–9.3 kPa, F2), advanced fibrosis (TE 9.4–13.9 kPa, F3 or F4), and cirrhosis (TE > 13.9 kPa, liver cirrhosis). Finally, fibrosis score such as AST-to-platelet ratio index (APRI) and Fibrosis-4 (FIB-4) are commonly used to assess the liver fibrosis stage. FIB-4 is calculated from age, AST, ALT and platelets, and APRI is calculated from AST and platelets. FIB-4 > 3.25 means advanced hepatic fibrosis (F3 or F4); APRI > 1.5 means significant liver fibrosis (F3 or F4). Our study defined Metavir stage F3 or F4 stage as advanced hepatic fibrosis diagnosis.

### Quality Assessment

In our study, two investigators individually assessed the quality of all included studies. The Agency for Healthcare Research and Quality (AHRQ) for cross-sectional studies were used to rate the risk of bias. The Newcastle-Ottawa Scale (NOS) were used to scale totally comprises subject selection, comparability of cases, and control as well as ascertainment of exposure in cohort study.

### Statistical Analyses

We used *I^2^* test to quantify the heterogeneity between studies. When *I^2^* statistic was above 50% and the corresponding *P* value was less than 0.05, pooled odds ratios (pooled ORs) were calculated with inverse-variance weighting using DerSimonian-Laird random effects models. Otherwise, fixed effects (inverse variance method, Mantel–Haenszel method and Peto’s method) will be performed. To find out the source of heterogeneity, we performed the stratified analysis based on two different measures of alcohol exposure (daily alcohol intake > 50 g and AUDIT-C ≥ 4), and three different populations (the first with only HIV infection, the second with HIV and HCV co-infection, and the third with a mixture of the above two). To evaluate the publication bias, we used Egger’s regression test to assess evidence for meta study effects (34). Meanwhile, sensitivity analysis was made to estimate the stability of individual studies to the overall effect. All the results of this meta-analysis were conducted on Stata 15.0 software. P-value less than 0.05 was defined as statistically significant.

## Results

### Literature Search

**Figure 1:**
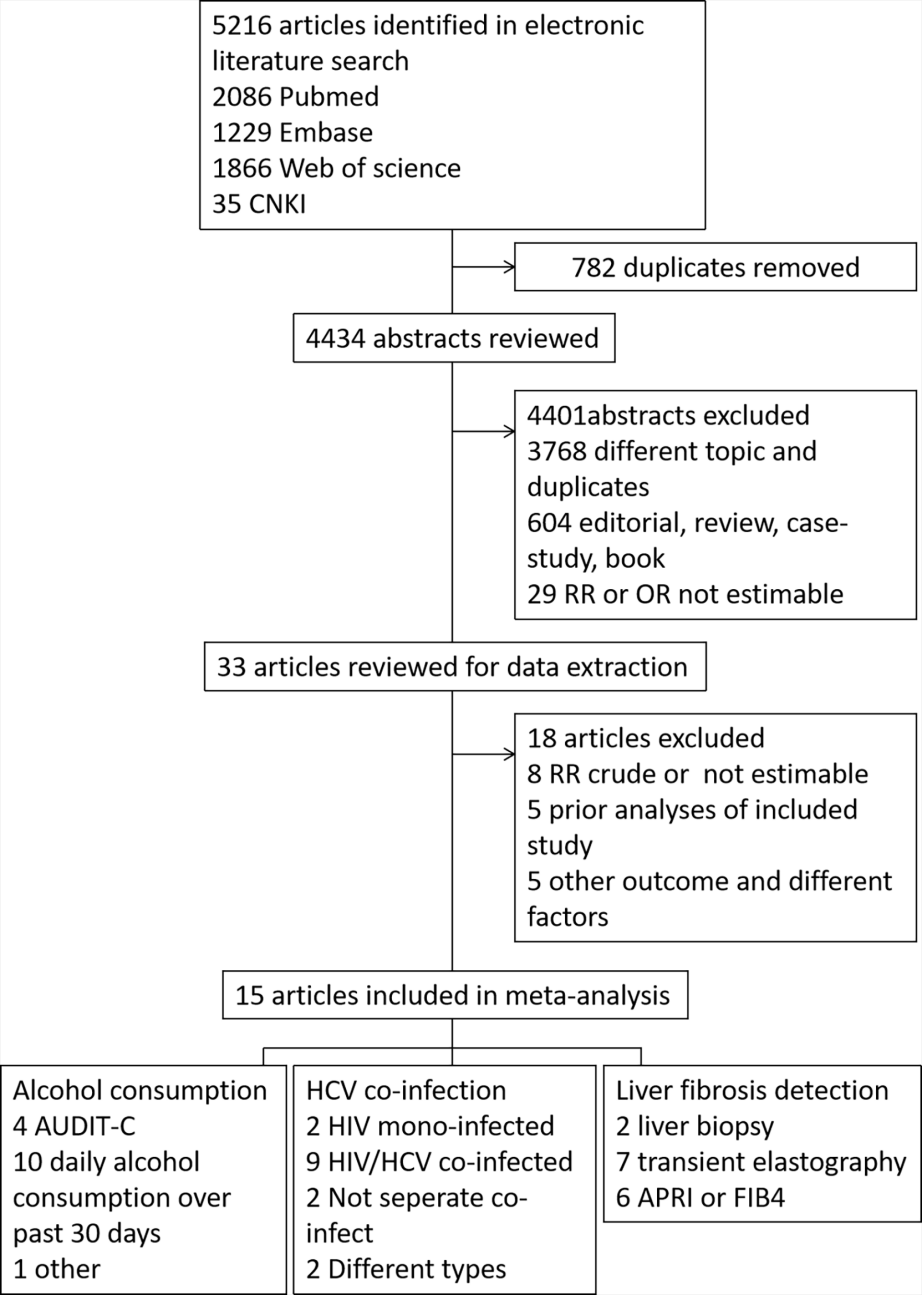
Flowchart of study selection.

The search results are summarized in **Figure 1**. A total of 5216 records were retrieved from Pubmed (2086 records), Embase (1229 records), Web of Science (1866 records) database, and CNKI database (35 records). Among all records, 782 were duplicates, and 4434 unique records were retained. After reviewing the title or/and abstract, 4401 records were further excluded, of which 3768 were excluded as they are not relevant, the article style excluded 604 records (review, meta-analysis and other style) and 29 records were excluded without Risk Ratio (RR) or OR estimable. The remaining 33 studies were downloaded for carefully screening, and 18 were excluded. Of the 18 excluded articles, eight studies didn’t estimate the RR or OR levels, five were prior analyses of included study, and five showed other outcomes or different factors. Therefore, 15 studies were included in the data analysis. The AHRQ score of the 12 cross-sectional articles was 8-10, as high quality. The NOS scale of the three cohort articles were high quality (7-8).

### Study Characteristics

**Table 1** displayed the main features of the studies included in this meta-analysis. The included studies comprised 22,676 individuals and 2,729 liver fibrosis cases. Three studies were set up as cohort studies (39, 42, 47) and the other twelve were cross-sectional studies. Eight studies were conducted in Europe, four in the USA, two in Africa, and one in South America. Among the included studies, 4 studies used the AUDIT-C questionnaire for screening alcohol use and ten studies used the history of alcohol intake per day as alcohol exposure assignment (one of which also used the AUDIT-C to quantify alcohol use). Only one study quantified the alcohol use by lifetime alcohol use. HIV and HCV co-infected accounted for a large proportion of the included studies, including nine articles. Only two articles have studied liver fibrosis in HIV mono-infected patients alone. Also, two studies did not separately analyze the effect of HCV on liver fibrosis in PLWH, and two studies included both HIV-HCV co-infection and HIV mono-infected types of patients. We separated the studies by the method of liver fibrosis detection, and there were two studies using liver biopsy, seven studies using transient elastography, and six studies using fibrosis score.

**Table 1:** Baseline data for the studies included in the analysis.

Author and year	Country	Type of study	Participants	Cases	Male %	Age	Co-infection	Exposure assignment	Outcome assignment	Adjustments
Schiavini, M., 2006 (35)	Italy	Cross-sectional retrospective analysis	110	31	82.7	29 (27-33)	HIV/HCV	Alcohol intake >50 g/day	Liver biopsy	None
Maria Cássia Mendes-Correa, 2008 (36)	Brazil	Cross-sectional retrospective analysis	234	168	74.3	39 (32-46)	HIV/HCV	Alcohol intake > 50 g/day for 6 months or longer any time	Liver biopsy	None
Merchante, Nicolas, 2010 (37)	Spain	Cross-sectional perspective analysis	258	29	67.0	42 (37-47)	HIV/HCV	Alcohol intake > 50 g/day	Transient elastography	Age
Pineda, J. A., 2010 (38)	Spain	Cross-sectional multicenter retrospective survey	1310	526	76	43 (39-46)	HIV/HCV	Alcohol intake > 50 g/day	Transient elastography	Gender, age, cannabis use, risk factor IDU, ART, years on ART, nadir CD4, CD4<200, HIV plasma load, years of HCV infection, HCV genotype, log HCV-RNA
Blanco, F., 2011 (9)	Spain	Cross-sectional retrospective analysis	681	Not mentioned	78.0	43 (37-49)	HIV-mono	Alcohol intake >60 g/day	Transient elastography	None
Collazos, J., 2011 (39)	Asturies, Northern Spain	Cohort study	1047	261	69.4	43.6 (39.7-47.2)	HIV/HCV	Alcohol abuse (>50 g/day during 5 years)	Transient elastography	Hepatitis B virus coinfection, Platelets, gender, g-Glutamyl transpeptidase
Cartón, J. A., 2011 (40)	Spain	Cross-sectional study	805	449	72.2	44.0 (39.7-47.4)	HIV/HCV	Alcohol intake >50g/day for≥5 years	Transient elastography	None
Auerbach, B. J., 2011 (41)	Uganda	Cross-sectional study	500	Not mentioned	33	38 (31-44)	mixed	Heavy liquor use (≥1.25 L/week), similarly for 160 g/day	Transient elastography	Age, gender, herb use, lifetime occupational fishing, HBsAg positive, HIV infection
Cartón, J. A., 2013 (42)	Spain	Cohort study	318	210	74.0	44 (40-48)	HIV/HCV	Alcohol intake > 50 g/day for 5 years	FIB-4 and APRI	None
Fuster, D.,2013 (43)	USA	Cross-sectional	Not mentioned	59	73.1	42.8 (35.5-50.1)	HIV/HCV	Total lifetime alcohol intake	FIB-4 and APRI	Sex, HCV RNA, CD4 count, age
Lim, J. K., 2014 (44)	USA	Cross-sectional study	2111	210	96.5	49 (43-55)	HIV-mono	ICD-9 diagnosis, AUDIT-c score≥4	FIB-4 and APRI	sex, race, BMI, diabetes, HBV infection, chronic HCV, and CD4 count and HIV RNA level (HIV-infected stratum).
Kim, H. N., 2017 (45)	USA	Cross-sectional study	12849	596	83	47 (38-51)	Mixed, HIV-mono, HIV/HCV	Hazardous: AUDIT-C score >=5 for men, >=4 for women	FIB-4 and APRI	Gender, race, HCV, HBV, Diabetes, current CD4 count, HIV viral load
Jaquet, A., 2017 (46)	West Africa	Cross-sectional study	807	43	29.4	43 (37-50)	Mixed	hazardous drinkers AUDIT-C≥3-4; heavy drinkers≥6	Transient elastography	Age, gender, referral hospital, antigen HBs, Anti-HCV, antiretroviral use
Yaya, I., 2018 (47)	France	Cohort study	1293	147	69.7	48 (44-51)	HIV/HCV	High risk>4 AU/day for men(>44-56 g/day) and >3 AU/day for women(>33-42 g/day)	FIB-4	None
Ferguson, T. F., 2020 (48)	USA	Cross-sectional study	353	Not mentioned	69	48.3 (38.3-48.3)	HIV/HCV, HIV mono	AUDIT-c score≥16 corresponds to a high risk of alcohol consumption; hazardous drinking as defined ≥40 g/day for females and ≥60 g/day for males	FIB-4 and APRI	age, sex, body-mass index, hepatitis B and HIV viral load.

### Alcohol Consumption and Liver Fibrosis Risk in PLWH

**Figure 2:**
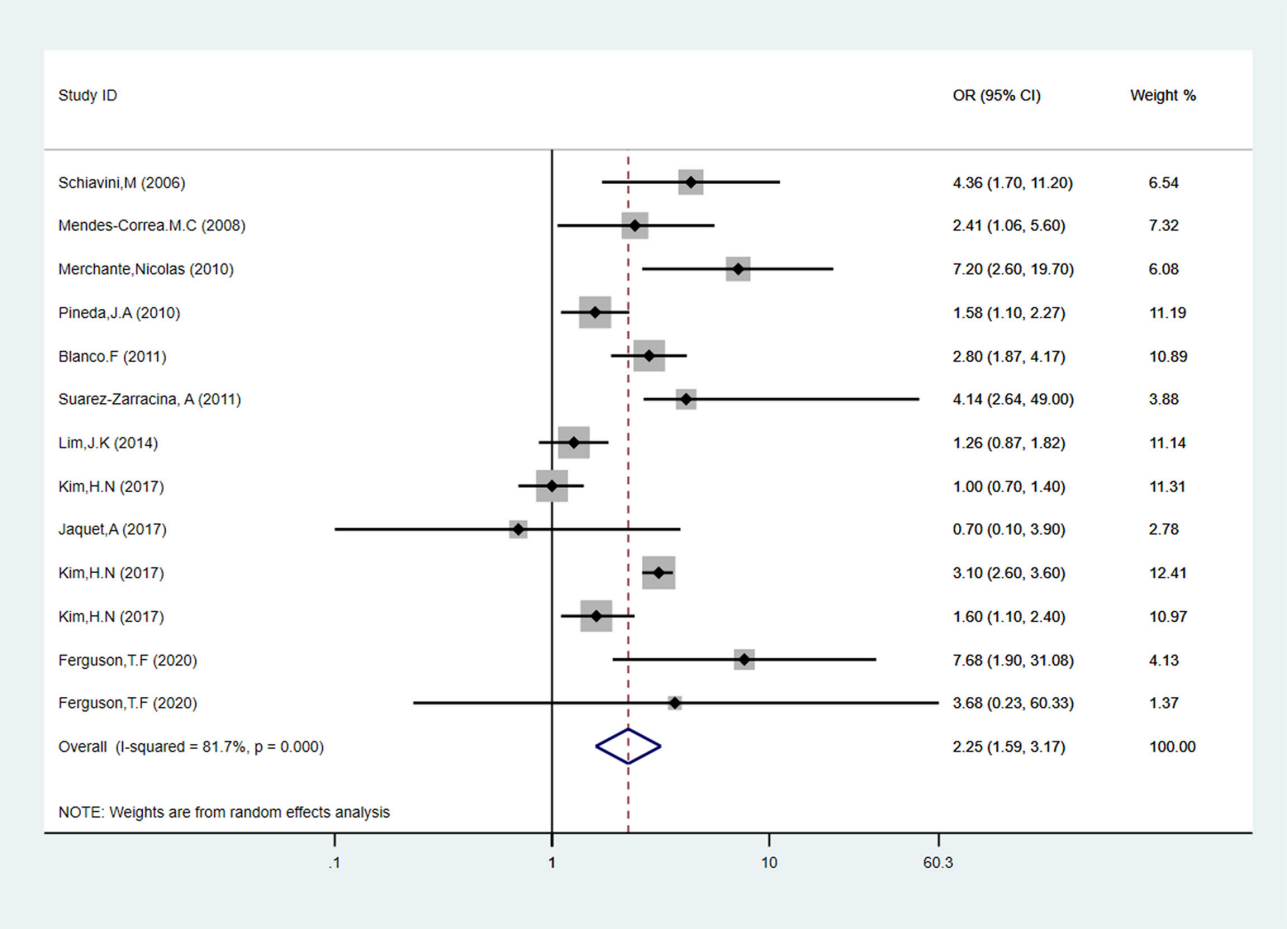
Forest plot of liver fibrosis pooled odds risk by alcohol consumption in cross-sectional studies.

Ten articles and thirteen data of all the fifteen studies evaluated alcohol consumption with the familiar way, comprising 2,052 liver fibrosis cases, and the details were shown in **Table 1**. As shown in **Figure 2**, in cross-sectional studies compared to people who with low-risk alcohol consumption, the PLWH with high-risk alcohol consumption had an increased risk of liver fibrosis, with pooled OR of 2.25 (95% CI: 1.59-3.17, p < 0.05). In cohort studies, compared to people who with low-risk alcohol consumption, the PLWH with high-risk alcohol consumption had an increased risk of liver fibrosis, with pooled OR of 2.89 (95% CI: 1.63-4.15, p = 0.796) showed in **Supplementary Figures 1** and **2**.

**Figure 3:**
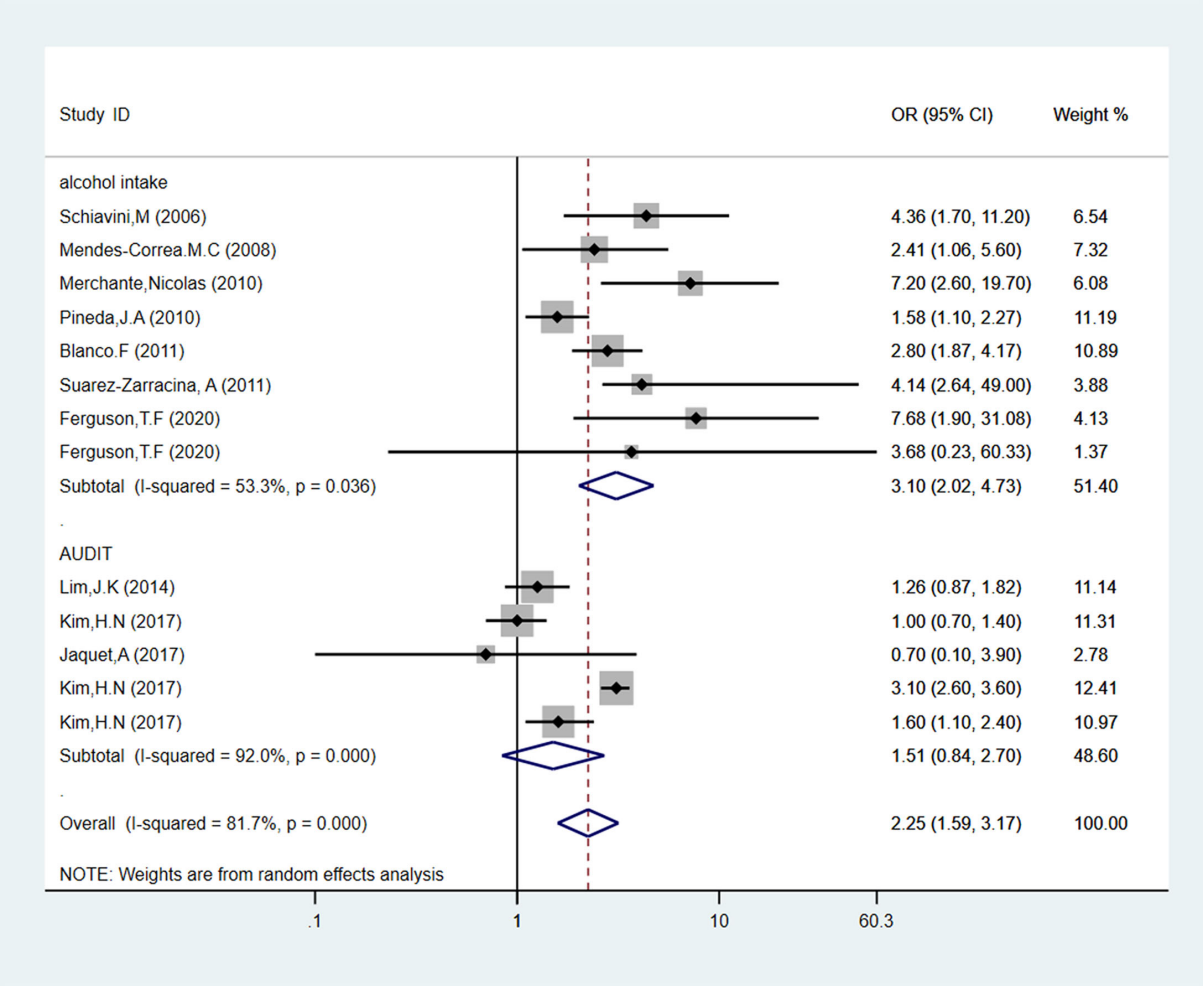
Forest plot of liver fibrosis pooled odds risk by alcohol consumption (sort by alcohol use determination) in cross-sectional studies.

Seven studies were evaluated daily alcohol consumption as “alcohol intake”, comprising 4,104 people. As shown in **Figure 3**, PLWH with a high level of alcohol consumption had an obvious increased risk of 3.10 (95% CI: 2.02-4.73, p < 0.05) for liver fibrosis than the low alcohol consumption patients. Also, three studies evaluated alcohol consumption by AUDIT-C questionnaire. Compared to the low-risk alcohol consumption patients, we found AUDIT-C score ≥ 4 is not a risk factor for liver fibrosis incidence in PLWH (pooled OR = 1.51, 95% CI: 0.84-2.70, p > 0.05).

**Figure 4:**
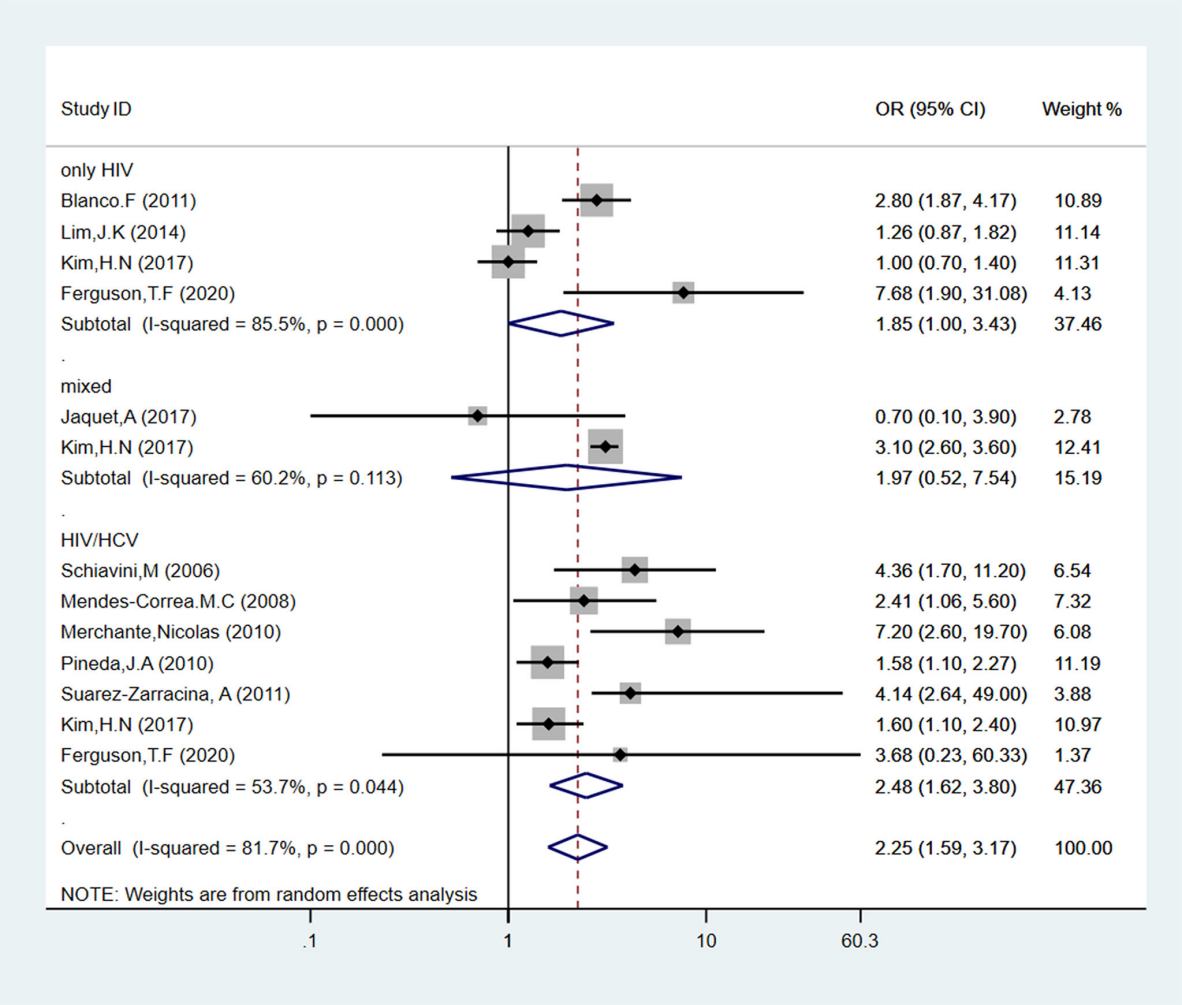
Forest plot of liver fibrosis pooled odds risk by alcohol consumption (sort by HCV co-infection or not) in cross-sectional studies.

According to the estimation of HCV co-infection, the studies can be divided into three sub-groups: HIV mono-infection, HIV and HCV co-infection, and was not separated the infection status (named as “mixed”). As shown in **Figure 4**, four studies analyzed the influence of alcohol intake in HIV mono-infection patients, reporting an increased association with high-risk alcohol consumption (pooled OR = 1.85, 95% CI: 1.00-3.43, p < 0.05). Seven studies, including 1799 participants, evaluated alcohol consumption and liver fibrosis incidence in patients with HIV and HCV co-infection. Compared to low-risk alcohol consumption patients, alcohol abused patients had an increased risk of 2.48 (95% CI: 1.62-3.80, p < 0.05) for liver fibrosis incidence. As for the mixed sub-group, there was no relationship between alcohol consumption and liver fibrosis (pooled OR = 1.97, 95% CI: 0.52-7.54, p > 0.05).

### Heterogeneity and Publication Bias

**Figure 5:**
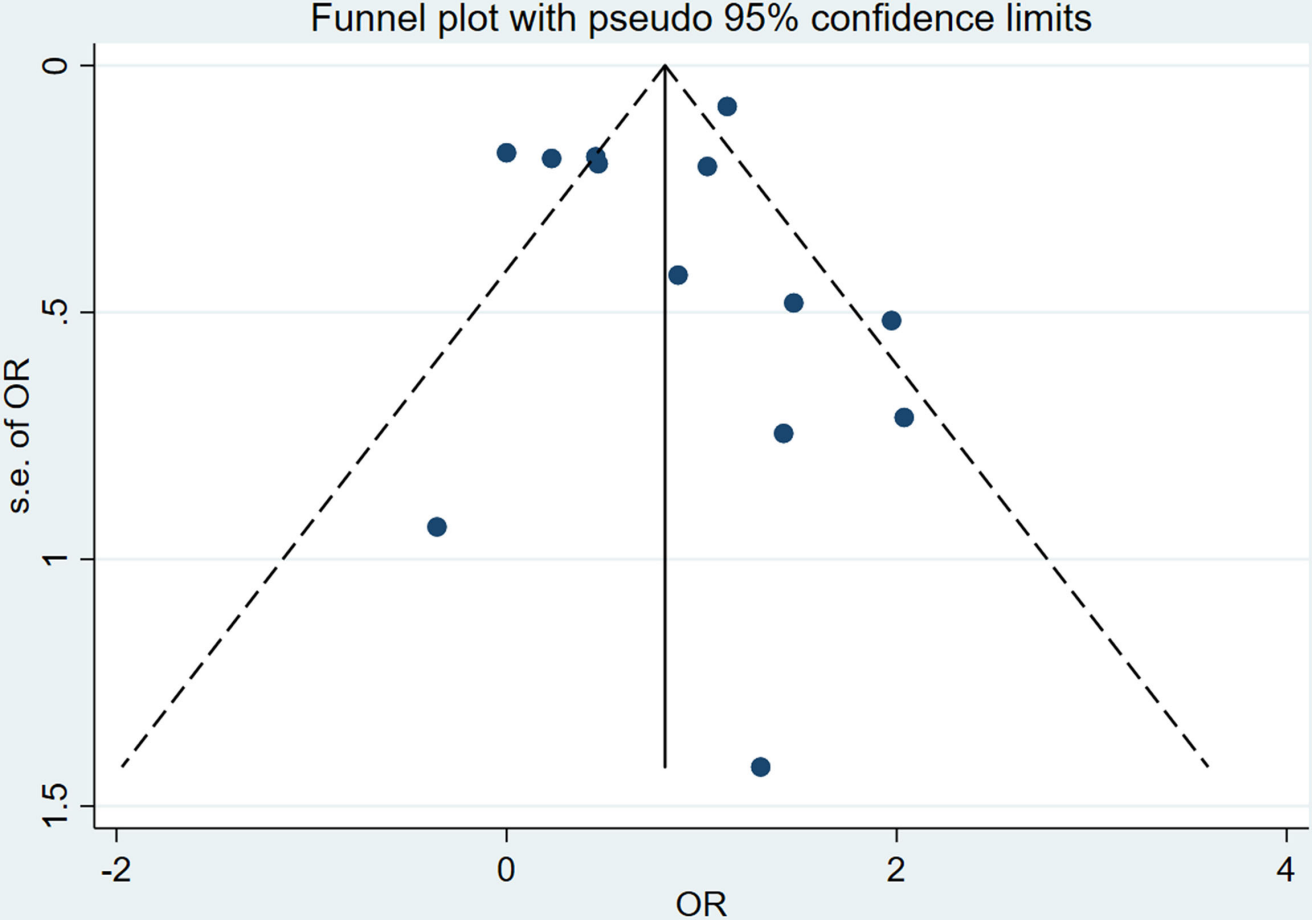
Funnel plot with pseudo 95% confidence limits for liver fibrosis.

The *I^2^* of total cross-sectional studies is 81.7%, which means the meta-analysis exist heterogeneity, so we choose the fixed effects model to analysis the data. The intuitive result of the funnel chart shows that the symmetry is general, as shown in **Figure 5**. The sensitivity analysis result in **Supplementary Figure 3** shows that the sensitivity of this study is low, and the results are more robust and credible. The Egger’s tests were performed to assess publication bias, and the results shown in **Supplementary Figures 2** and **4** that there was no publication bias.

## Discussion

Our findings indicate that high-risk alcohol drinking is a risk factor for liver fibrosis for PLWH, especially HIV and HCV co-infected patients. Most importantly, our meta-analysis shows different patterns in subgroups: for different alcohol consumption groups, we consider the daily alcohol consumption is more sensitive for analysis of the effect of alcohol abuse rather than AUDIT-C score; for HCV co-infected or not subgroups, in HIV and HCV co-infected patients, high-risk alcohol consumption has a higher risk of liver fibrosis than HIV mono-infected patients. Although only three included studies used a cohort study approach with a high level of evidence, their results showed that alcohol exposure is a risk factor for liver fibrosis in people infected with HIV, regardless of co-infection with HCV (12, 36, 49).

Recently published meta-analyses have provided information about alcohol abuse as a risk factor for liver cirrhosis in general people (50). Our meta-analysis confirms previous findings in PLWH and provides additional value with five distinct subgroup analyses. In this study, strict inclusion and exclusion criteria were formulated according to the research purpose, and studies with significant differences in the definition of high-risk alcohol drinking were excluded for analysis (41, 43). In addition, according to the full consideration of the risk of liver fibrosis caused by the drinking of HIV-infected persons in different regions, the population characteristics, and the diagnose methods are different in each research inclusion.

According to our data, the alcohol intake determination pathway may influence the risk of alcohol consumption, which could have impacted our findings. One study noted that a stepwise increased risk of advanced liver fibrosis with greater severity of alcohol use (51). They also demonstrate that all alcohol use categories are strongly associated with advanced hepatic fibrosis in HIV/HCV-coinfected patients. Our study found that daily alcohol intake>50 g was a significant risk factor for F3 or F4 stage liver fibrosis. Although the US AUDIT has adapted the WHO AUDIT to a 14 g standard drink (52), AUDIT-C score ≥ 4 (similarly to ≥ 52 g/day) were not risk factors for liver fibrosis incidence in PLWH in our study. Also, we found there were studies (43) using a structured interview to recall patterns of total lifetime alcohol intake, which may be better-predicted liver injury than current heavy alcohol use. But this method is rarely applied because it is not suitable for large sample research and the accuracy deserves further investigation. Serum phosphatidylethanol (PEth) is a biological marker that reflects alcohol use within an approximate 3–4-week period. Although PEth does not rely on self-report by participants, the period of alcohol determination is too short of reflecting the truth status of the alcohol consumption (48). The results of AUDIT-C show their cut-offs produce a high rate of false positives resulting largely from not measuring alcohol consumption correctly (52). However, the daily alcohol consumption can precise distinct high level of alcohol consumption. We recommend referring more to the results of daily alcohol consumption rather than the AUDIT-C as the measure of alcohol intake, for several reasons that can be summarized as follows. Initially, AUDIT-C is suitable for detecting early drinkers rather than long-term drinkers, and liver fibrosis is a chronic process, so alcohol measurement using AUDIT-C may not be a good guide for liver fibrosis. Secondly, AUDIT-C investigates not only the amount and frequency of alcohol consumption, but also the severity of adverse health conditions that may be caused by alcohol (53), so AUDIT-C may have introduced other non-alcoholic factors in the study of the relationship between alcohol consumption and liver fibrosis, making the study of this association inaccurate. In the general population, both AUDIT-C and daily alcohol consumption are applicable; but in sensitive populations such as HIV infection, using daily alcohol consumption is more accurate.

The result, which was divided according to the different study populations, showed that the combined effect size was similar to the total effect in the three subgroups, and the effect was not statistically significant only in the mixed group that did not distinguish HCV infection status. In the subgroup infected with HIV only, there was an increased risk for alcohol abuse compared to no hazard alcohol consumption. In the subgroup co-infected with HCV, the OR was 2.48. In the mixed subgroup, the pooled OR was 1.97. A cross-sectional study (54) by Kim showed that the risk of liver fibrosis from alcohol exposure was higher in those co-infected with HIV and HCV relative to those infected with HIV only (1.60 vs. 1.00), and that risk was the highest in the mixed group. While another cross-sectional study (55) by Ferguson presented a contrary conclusion, it implied that those infected with HIV only had a higher OR than those co-infected with HIV and HCV (7.68 vs. 3.68). Co-infected with HCV can definitely influence liver health status among PLWH (42), but a more rigorously designed cohort study is needed to confirm the effect of alcohol.

HCV infection is an independent risk factor for liver fibrosis (56). HIV and HCV share the same routes of virus transmission. According to the previous study, the co-infection ratio of HIV and HCV was about 10% of patients with HIV in the western world (57). Previous reported that in China, the highest HIV/HCV prevalence was in the Central region (28.2% of PLWH) (58). HCV has the same infection route as HIV, especially injecting drugs, so the HIV-HCV co-infection rate of injecting drug users (IDUs) is much higher than that of other populations. In a study of 359 IDUs with HIV infection by A-Mei Zhang et al., the HCV co-infection rate was as high as 45.68% (2). Over two-thirds of patients with HIV-HCV co-infection had moderate-to-significant liver fibrosis as measured by either APRI or FIB4 in China (36). In other countries, the liver fibrosis rate in HIV-HCV co-infected patients is 30%-50% (59, 60) detected by transient elastography. HIV/HCV co-infected patients were more likely to have liver fibrosis compared with HIV or HCV mono-infected (61). Patients infected with HIV-1 or HCV or who drink alcohol exhibit metabolic abnormalities that cause changes in the levels of n-acetylaspartate, choline, and creatine in different regions of many organs, including the brain and liver (62). Treatment of HIV/HCV coinfection in alcohol users is complicated by drug-drug interactions and the effects of alcohol on drug metabolism (63). But other researchers found liver fibrosis progresses faster in HIV mono-infection, other than in HIV/HCV co-infection (64). Direct-acting antivirals (DAA) lead to a decreased risk of HCV progression. After DAA treatment, HIV/HCV co-infected patients had similar risk of liver-related deaths and events but had a higher risk of all-cause and non-liver-related deaths and non-liver-related cancers compared with HCV mono-infected individuals (65). Although DAA treatment can successfully increase HCV cure rates with minimal toxicity in HIV/HCV patients (66), there are few cohort studies on liver fibrosis and other diseases in HIV/HCV co-infected patients receiving DAA for several years. The delayed effect of liver fibrosis and disease should be continually followed in the future study.

There are some limitations of this review. First, the number of the included articles and the contributions’ quality were limited. Only three studies in our study are cohort studies and cannot be analyzed. Also, we were unable to investigate in detail the role of many study design characteristics, such as adjustment for potential confounders, the length of alcohol abuse, and others that may play a role in the development of liver fibrosis, because of the small number of studies published. Second, although self-reported alcohol consumption is generally reliable, it may result an underestimate with the actual consumption of alcohol. Third, different studies adopting different diagnose criteria will cause differences between studies. Although it is generally accepted that the diagnosis of liver fibrosis is 4≥F≥3, the application of different detection methods will have certain errors in the diagnosis of liver fibrosis. Fourth, the heterogeneity within studies is very high, so we used a random effects model to analysis the data.

As for clinical use, it is necessary to elucidate the role of other risk factors, such as genetic vulnerability, gender, metabolic risk factors, and drinking patterns over the life course, in high-quality research. Also, alcohol abuse should be avoided in PLWH, especially in HIV and HCV co-infected patients. Patients drinking at high levels should regularly receive inspections of liver fibrosis-related indicators and related interventions to reduce their intake. If necessary, patients should be treated with hepatoprotective drugs. For doctors, when prescribing antiviral treatments for HIV-infected patients with alcohol abuse, hepatotoxicity and metabolic toxicity of the drugs should be fully considered, and therapeutic drugs with less hepatotoxicity should be used as much as possible.

## Conclusion

This study reported that high-risk alcohol consumption is a risk factor of liver fibrosis for PLWH, especially for HIV/HCV co-infected patients. For different alcohol consumption subgroups, we considered the daily alcohol consumption is more sensitive for analysis of the effect of alcohol abuse rather than AUDIT-C score. Inconsistent definitions of alcohol exposure can lead to different conclusions. As for whether HCV co-infected subgroups, high-risk alcohol consumption has a higher risk of liver fibrosis in HIV and HCV co-infected patients rather than HIV mono-infected patients. Hepatotoxicity drugs should be considered by doctors when prescribing antiviral treatments for HIV-infected patients with alcohol abuse, or co-infected with HCV.

## Data Availability Statement

The original contributions presented in the study are included in the article/**Supplementary Material**. Further inquiries can be directed to the corresponding author.

## Author contributions

Conceptualization: HL and YZ. Methodology: HL and YZ. Software: HL and HT. Validation: HL and YL. Formal analysis: SH, YW, and YZ. Data curation: YL. Writing—original draft preparation: HL and HT. Writing—review and editing: HL and YZ. Visualization: HL. Supervision: YZ. Project administration: HL and YZ. Funding acquisition: YZ. All authors contributed to the article and approved the submitted version.

## Conflict of Interest

The authors declare that the research was conducted in the absence of any commercial or financial relationships that could be construed as a potential conflict of interest.

The handling editor WT has declared a past co-authorship with one of the authors YZ.

## Publisher’s Note

All claims expressed in this article are solely those of the authors and do not necessarily represent those of their affiliated organizations, or those of the publisher, the editors and the reviewers. Any product that may be evaluated in this article, or claim that may be made by its manufacturer, is not guaranteed or endorsed by the publisher.

## Abbreviations

PLWH, people living with HIV; pooled OR, pooled odds ratio; CI, confidence interval; HBV, hepatitis B virus; HCV, hepatitis C virus; ROS, reactive oxygen species; ECM, extracellular matrix; AUDIT-C, The Alcohol Use Disorders Identification Test; TE, transient elastography; APRI, AST-to-platelet ratio index; FIB-4, Fibrosis-4; AHRQ, The Agency for Healthcare Research and Quality; NOS, The Newcastle-Ottawa Scale; RR, Risk Ratio; OR, odds ratios; PEth, phosphatidylethanol; IDU, injecting drug user; DAA, Direct-acting antivirals.
