# Supplementary material for: Alcohol Consumption and Risk of Liver Fibrosis in People Living With HIV: A Systematic Review and Meta-Analysis
Source: Front Immunol. 2022 Mar 18;13:841314. doi: 10.3389/fimmu.2022.841314 (PMC8971654; doi:10.3389/fimmu.2022.841314)
Supplement: Supplementary file 1 [file DataSheet_1.docx]

Supplement figures

Alcohol consumption and risk of liver fibrosis in HIV patients: a systematic review and meta-analysis

Hang Lyu, et al.


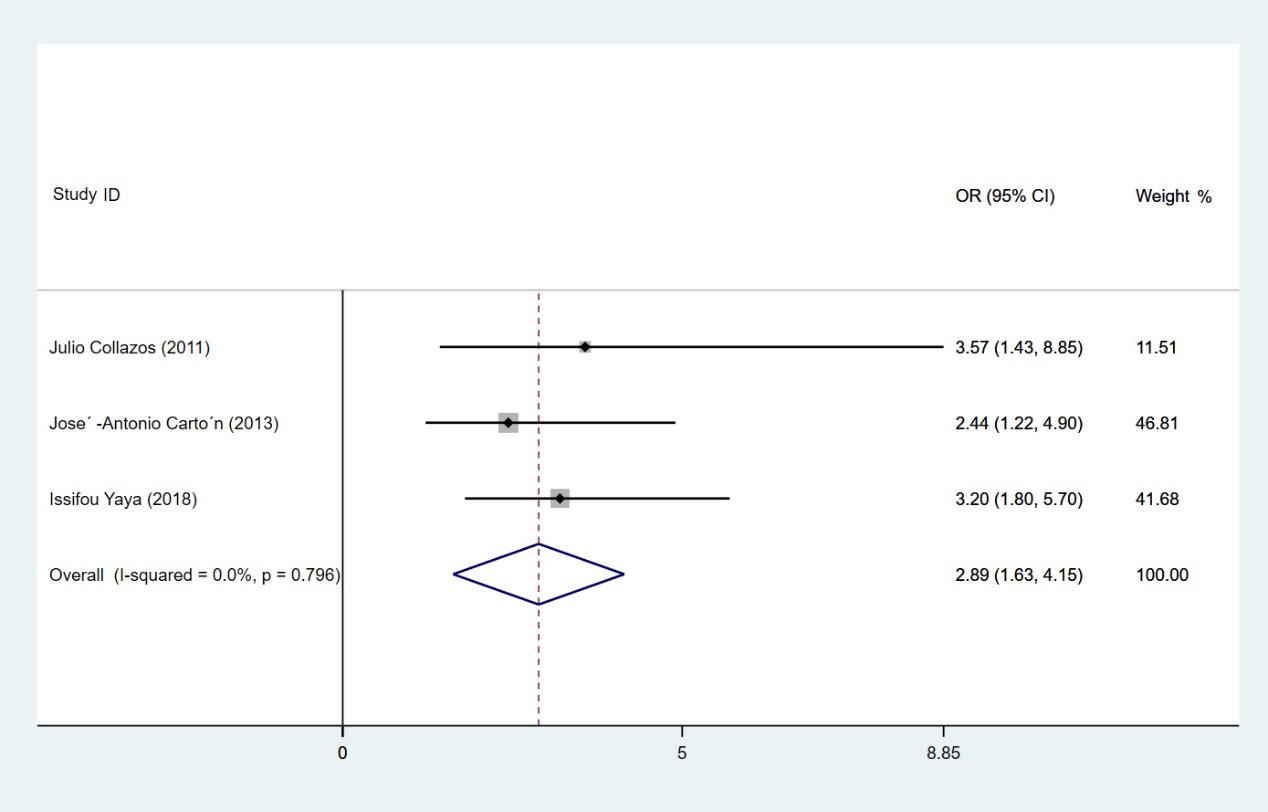


Supplement figure1. Forest plot of liver fibrosis pooled odds risk by alcohol consumption in cohort studies.


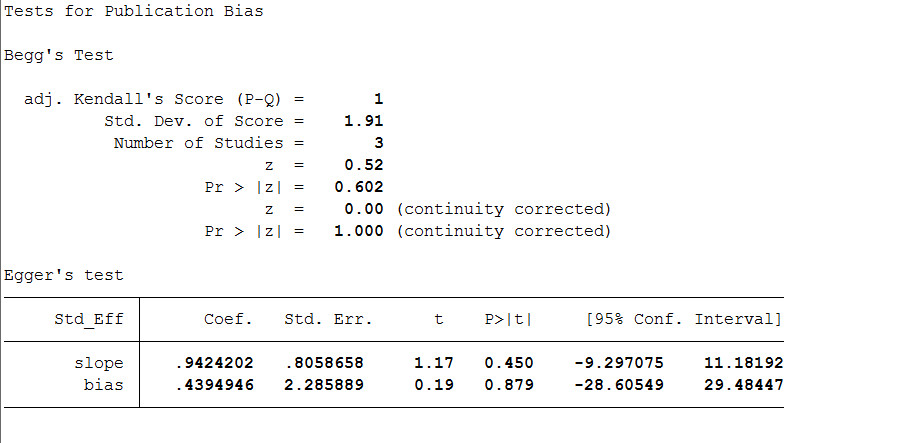


Supplement figure2. Egger’s test for cohort study analysis.


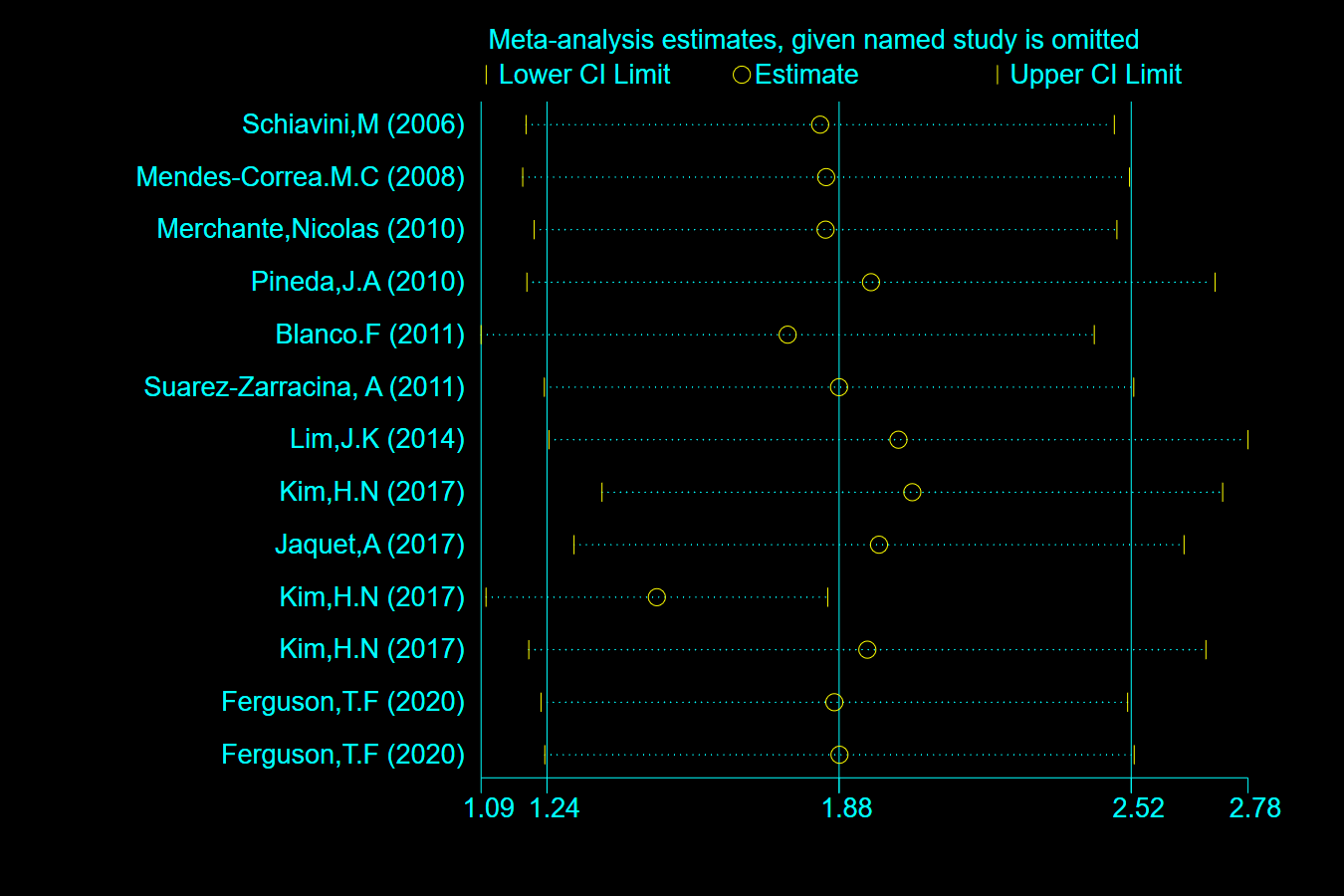


Supplement figure3. Results of sensitivity analysis


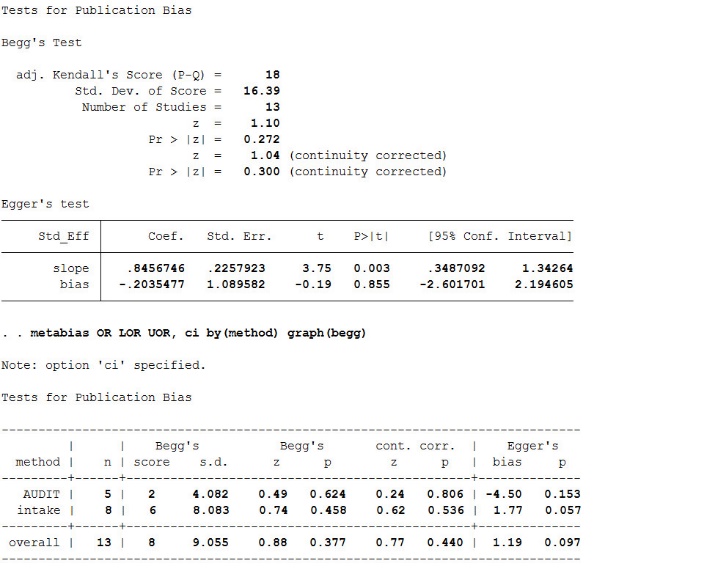


Supplement figure4. Egger’s test of cross-sectional study analysis.
